# Development of Thermostable Lyophilized Inactivated Polio Vaccine

**DOI:** 10.1007/s11095-014-1359-6

**Published:** 2014-04-24

**Authors:** Heleen Kraan, Paul van Herpen, Gideon Kersten, Jean-Pierre Amorij

**Affiliations:** 1 Institute of Translational Vaccinology (Intravacc), Antonie van Leeuwenhoeklaan 9, P.O. Box 450, 3720 AL Bilthoven, The Netherlands; 2 Division of Drug Technology, Leiden Academic Center for Drug Research, Leiden University, Leiden, The Netherlands

**Keywords:** design of experiments, formulation, inactivated polio vaccine, lyophilization

## Abstract

**Purpose:**

The aim of current study was to develop a dried inactivated polio vaccine (IPV) formulation with minimal loss during the drying process and improved stability when compared with the conventional liquid IPV.

**Methods:**

Extensive excipient screening was combined with the use of a Design of Experiment (DoE) approach in order to achieve optimal results with high probability.

**Results:**

Although it was shown earlier that the lyophilization of a trivalent IPV while conserving its antigenicity is challenging, we were able to develop a formulation that showed minimal loss of potency during drying and subsequent storage at higher temperatures.

**Conclusion:**

This study showed the potential of a highly stable and safe lyophilized polio vaccine, which might be used in developing countries without the need of a cold-chain.

**Electronic supplementary material:**

The online version of this article (doi:10.1007/s11095-014-1359-6) contains supplementary material, which is available to authorized users.

## Introduction

Poliomyelitis is a highly infectious disease, which mainly affects young children. The disease, caused by any one of three serotypes of poliovirus (type 1, type 2 or type 3) has no specific treatment, but can be prevented through vaccination.

Currently, the live attenuated oral poliomyelitis vaccine (Sabin OPV) is the vaccine of choice to prevent polio outbreaks and stop transmission of wild polioviruses, especially in the remaining endemic countries. However, a major concern is the ability of OPV to revert to a form that can cause paralysis, so-called vaccine-associated paralytic poliomyelitis (VAPP). Permanent use of OPV would continue to generate circulating vaccine-derived polioviruses (cVDPVs) that will inevitably lead to new outbreaks (1). Therefore, the new endgame strategy of the Global Polio Eradication Initiative (GPEI) includes the introduction of an inactivated polio vaccine (IPV) into all routine immunization programs followed by phased withdrawal of OPV (2). In most of the high-income countries, IPV based on Salk strains is already the present preferred way to eliminate the risk of VAPP and cVDPVs.

To achieve global polio eradication, an (improved) IPV must be efficacious, inexpensive, safe to manufacture, and easy to administer (3). The feasibility of current IPV in developing countries is limited, because IPV is more expensive than OPV and is administered through injections only. In order to extent the availability of IPV, the World Health Organization (WHO) and the Institute for Translational Vaccinology (Intravacc) in the Netherlands have developed a non-commercial IPV for technology transfer to developing countries (4). Because the containment of the wild-type Salk poliovirus during production might be an issue, especially in developing countries, the new vaccine will be based on the traditional Sabin OPV strains (sIPV). For reduction in costs, Intravacc is developing sIPV formulations that show dose sparing by using an adjuvant (5) and/or other immunization routes (6,7).

Vaccine delivery encompasses both administration of the vaccine formulation to specific sites and delivery of the antigen to and activation of relevant cells of the immune system (8). Since alternative delivery methods and improved vaccine formulations have the potential to make vaccine delivery easier and safer (9,10), several alternative vaccine delivery methods are currently being developed. However, the focus in vaccine development has been on optimization of the immunological properties, while stability issues are minimally addressed. Most vaccines, IPV included, are insufficiently stable to allow them to be purified, transported and stored at unrefrigerated conditions (11,12).

One way to improve the storage stability of (s)IPV might be conversion into the dry state as is known to improve the stability of biopharmaceuticals (13). An increased shelf life is not only of use for the final product for use within 3 months to 2 years, but also for stockpiling (1–10 or more years of storage). Particularly after polio eradication, a stockpile of polio vaccines is required to anticipate the potential risk of new polio outbreaks caused by circulating VDPV (even after OPV cessation) (14,15) or bioterrorism attacks. In order to achieve an optimal vaccine stockpile, various issues need to be considered. The shelf life is paramount, because a delayed expiration time will reduce the stockpile costs (16). Moreover, storage of dried materials at ambient temperature, including concomitant costs (e.g., reformulation costs), is cost effective compared with storage options at low temperatures. In addition, the ability to develop solid antigen formulations is crucial for new vaccine delivery routes including dermal delivery by coated or dissolving microneedles, parenteral delivery by powders or dissolvable needles, and pulmonary delivery of powders (8). Technologies for producing dried biologicals include vacuum drying, air-drying, coating, spray (freeze-)drying and foam-drying (17–19). One of the oldest and commonly used techniques is freeze-drying, also called lyophilization. However, during the lyophilization process the proteinaceous vaccine is subjected to freezing and drying stress by which its activity can be lost. Therefore, cryoprotective and lyoprotective agents are required. Many compounds, such as sugars, polymers, amino acids and surfactants, have been shown to improve the stability of biopharmaceuticals during lyophilization and subsequent storage (20).

The aim of current study is to design IPV in the dry state with maintenance of the potency. A potency indicating parameter is D-antigenicity, which can be determined in ELISA using specific antibodies as stated in the European Pharmacopeia. Lyophilization of polio vaccines has been shown to be challenging since earlier studies failed to obtain a stable product with preservation of all three serotypes (21–23). We describe the development of an IPV formulation by selecting excipients that i) minimize potency loss upon drying and subsequent reconstitution, and ii) increase the stability of IPV at elevated temperatures. Extensive excipient screening was combined with the use of a Design of Experiment (DoE) approach in order to achieve optimal results with high probability.

## Materials and Methods

### Materials

Trivalent IPV, containing the inactivated Mahoney strain for type 1, MEF for type 2 and Saukett for type 3, was obtained from the process development department of Intravacc (Bilthoven, The Netherlands). The ten times concentrated trivalent bulk used in this study was determined at a nominal concentration of 400-80-320 DU/ml by ELISA as described (24).

The excipients sucrose, D-sorbitol, D-trehalose dihydrate, mannitol, L-glutamic monosodium salt monohydrate (MSG), glycine, myo-inositol, magnesiumchloride hexahydrate, lithium chloride and ovalbumin were all purchased from Sigma (St. Louis, MO). Peptone (vegetable) and dextran (6 kDa, from *Leuconostoc* ssp.) were from Fluka (Buchs, Switzerland) and sodium chloride was from Merck (Darmstadt, Germany). To prepare 10 mM McIlvaine buffer, 10 mM citric acid (Sigma-Aldrich, St. Louis, MO) was added to 10 mM disodium hydrogen phosphate dehydrate (Na_2_HPO_4_) (Fluka, Buchs, Switzerland) in a ratio of 1:6 resulting in a pH-value of 7.0. All excipients used were of reagent quality or higher grade.

### Methods

#### Dialysis

Unless otherwise indicated, the trivalent IPV bulk material was dialyzed against 10 mM McIlvaine buffer (pH 7.0) using a 10 kDa molecular weight cut-off, low-binding regenerated cellulose membrane dialysis cassette (Slide-A-Lyzer®, Pierce, Thermo Scientific, Rockford, IL) to replace the buffer components of the IPV bulk (M199 medium).

#### Formulations

All excipients were dissolved in McIlvaine buffer at a double concentration of the indicated end concentration. The dialyzed IPV was mixed 1:1 by volume with the formulation to be tested. Subsequently, 2 ml neutral glass injection vials (Müller + Müller, Holzminden, Germany) were filled with 0.2 ml of the IPV-excipient mixtures and half-closed with 13 mm pre-dried (overnight at 90°C) rubber stoppers (type V9250 from Helvoet Pharma, Alken, Belgium).

#### Drying Processes

The filled (0.2 ml/vial) and half-stopped vials were loaded into a pilot freeze dryer (freeze-drying unit sublimator 2-3-3, Zirbus) at a shelf temperature of −50°C, or at a shelf of 4°C and subsequently frozen to −50°C by reducing the shelf temperature at a rate of 1°C/min. These different processes will be denoted as fast and slow freezing, respectively. The vials were kept at a temperature of −50°C for 2 h. For the primary drying phase, the shelf temperature was increased at a rate of 0.2°C/h to −45°C (while decreasing the chamber pressure to 0.045 mbar) followed by drying for 3 h. The secondary drying phase was performed by further increasing the shelf temperature at 0.02°C/min to 25°C while decreasing the chamber pressure to 0.01 mbar, followed by 24 h drying at 25°C. At the end of the cycle, the vials were closed under vacuum, sealed with alu-caps and kept at 4°C until analysis.

In literature, different vacuum drying processes are described (25–27). The vacuum drying process used in current study was slightly adapted, due to the characteristics of IPV. Briefly, the vials were loaded at shelves of 15°C and kept at that temperature for 10 min. The chamber pressure was reduced to 1 mbar in different ramping steps of 15 min and starting at a 25 mbar chamber pressure. The temperature was decreased to −10°C for 1 h at 0.05 mbar and for 1 h at 0.03 mbar so that freezing of the formulations was prevented since product temperature was kept above the ice-nucleation temperature of the formulations. Subsequently, shelf temperature was increased at 0.05°C/min to 30°C. At the end of the cycle, the vials were closed under vacuum, sealed with alu-caps and kept at 4°C until analysis.

#### Design of Experiments (DoE)

The Design of Experiments models for D-antigen recoveries measured by ELISA were evaluated in Modde 9.1 software (Umetrics AB, Umea, Sweden) to establish the stability profiles of the lyophilized IPV. In a first pilot experiment, the effect of common used stabilizers, i.e., sucrose (0–20% w/v), trehalose (0–20% w/v), mannitol (0–10% w/v), dextran (0–10% w/v) and NaCl (0–63 mM), was determined using a D-optimal design containing of 22 different formulations and three replicates of the center point (supplemental data, Table SI). For the screening of some excipients, a full factorial DoE was performed around sorbitol, MgCl_2_, monosodium glutamate (MSG) and mannitol, all within the concentration range from 0 to 10% w/v. In this full factorial design 2^4^ formulations were tested and three replicates of the center point (Table II). For the optimization experiment a central composite circumscribed (CCC) design was used around sorbitol (8 to 12% w/v), MgCl_2_ (5 to 12% w/v) and MSG (5 to 12% w/v). The CCC design consisted of eight corner experiments, six axial experiments and three replicated center points (Table III). The models were fitted using partial least squares (PLS) regression and subsequent optimized by deleting non-significant terms leading to a model with the best model performance parameters, i.e., goodness of fit (R^2^), goodness of prediction (Q^2^), model validity and reproducibility.

#### D-antigen ELISA

A sandwich enzyme-linked immunosorbent assay (ELISA) was used to quantify D-antigen units (DU) of the lyophilized polio vaccine formulations as described by Ten Have *et al.* (2012) (28). Briefly, microtiter plates were coated with serotype-specific bovine anti-polio serum (Bilthoven Biologicals, Bilthoven, The Netherlands). After washing dilutions of IPV-formulations were added (in duplicate). After a 30 min incubation at 37°C under gentle shaking, plates were washed and a mixture of serotype-specific anti-poliovirus monoclonal antibody (mab 3-4-E4 (type 1), 3-14-4 (type 2), 1-12-9 (type 3); all from Intravacc, Bilthoven, The Netherlands) and HRP-labeled anti-mouse IgG (GE Healthcare, Buckinghamshire, UK) was added and incubated for 30 min at 37°C while shaking. Subsequently, plates were washed followed by addition of ELISA HighLight signal reagent (ZomerBloemen BV, Zeist, The Netherlands). Chemiluminescence was measured for 10–15 min by using a luminometer (Berthold Centro LB960). The signal at maximum intensity was used to calculate D-antigen content relative to the reference standard. Unless otherwise indicated, DU recovery values were shown as normalized values for liquid formulations prior to lyophilization.

#### Dynamic Light Scattering (DLS)

Particle size measurements were performed using a Zetasizer Nano-ZS system (Malvern Instruments, Malvern, UK). DLS measurements were done in triplicate with 0.5 ml liquid (undialyzed) IPV bulk at an operating temperature of 25 ºC. Homogeneity of the size distribution was reflected in the polydispersity index (PdI), which ranges between 0.0 (fully homogeneous size distribution) and 1.0 (random size distribution).

#### Moisture Content Analysis

The water content was determined using a Karl Fischer coulometric titrimeter (Model CA-06 Moisture meter, Mitsubishi). The samples were weighted, subsequently reconstituted in the Karl-Fischer reagent, Hydrana Coulomat A (Fluka, Buchs, Switzerland), and injected into the titration vessel. Each vial was measured in triplicate. The empty vials were weighted and the water content was calculated based on the water content measured by the titrimeter, the weight of the lyophilized product in the vial, the reconstitution volume of the reagent, titration volume and the water content of the blank titration.

#### Differential Scanning Calorimetry (DSC)

The thermodynamic behavior of the formulations was determined by differential scanning calorimetry (DSC). Aluminum DSC pans were filled with the liquid formulations and subjected to a controlled temperature program in a differential scanning calorimeter (DSC Q100, TA Instruments). The samples were cooled to −70°C at a rate of 10°C/min, kept isothermal for 2 min, and subsequent heated to 20°C at a heating rate of 10°C/min. The sample chamber was purged with nitrogen gas (50 ml/min). The glass transition temperatures (T_g_’) were determined as the midpoint of the discontinuities in the heat flow curves using thermal analysis software (Universal Analysis 2000, TA Instruments).

## Results & Discussion

### Common Lyoprotectants

The first design of experiment approach was based on the most commonly used lyoprotectants, i.e., sucrose, trehalose and mannitol, in combination with dextran, with and without NaCl (Table SI, supplemental data). These excipients are known to provide physical and biochemical stabilization as well as appropriate structural properties to the cake structure, during and after lyophilization (29–32).

**Fig. 1:**
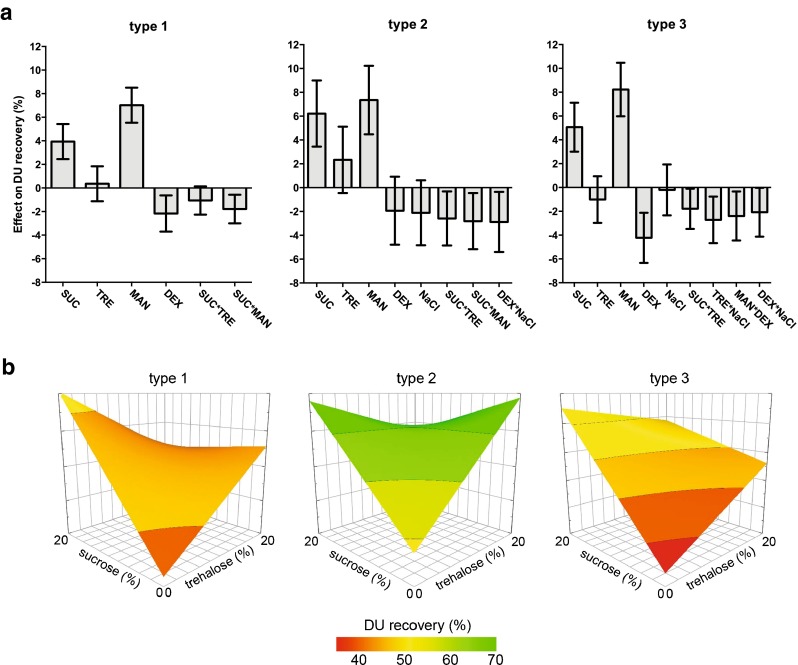
Stabilizing potential of the excipients sucrose (SUC), trehalose (TRE), mannitol (MAN), dextran (DEX) and NaCl on DU recovery directly after lyophilization. Main and interaction effects that contribute (per serotype) to the best model, according to the model performance parameters (Q^2^ = 0.650, R^2^ = 0.905 (type 1); Q^2^ = 0.592, R^2^ = 0.873 (type 2); Q^2^ = 0.671, R^2^ = 0.929 (type 3)), are depicted in coefficient plots (**a**). Surface response plots of the DU recovery for each serotype based on formulations containing sucrose and trehalose in combination with 10% mannitol (without dextran or NaCl) (**b**).

A partial least squares (PLS) regression model was fitted and optimized per serotype, which resulted in valid models to predict the DU recoveries directly after lyophilization according to the model performance parameters. For serotype 1, 2 and 3, the Q^2^ values were, respectively, 0.650, 0.592 and 0.671, while the R^2^ values were 0.905, 0.873 and 0.929. The effects of the different stabilizers, after optimization (excluding non-significant parameters) on the DU recovery after lyophilization are presented in Fig. 1a.

Without the addition of stabilizers, DU recoveries of type 1, 2 and 3 after lyophilization were 9, 11 and 2%, respectively (Table SI, supplemental data). Both sucrose and mannitol are able to stabilize all serotypes to a certain extent during the lyophilization process (Fig. 1a). Dextran has a negative effect on type 1 and 3 during the lyophilization process, whereas the addition of NaCl has no significant effect on the DU recovery, independent of serotype (Fig. 1a). Best results, with recoveries of approximately 55%, 85% and 50% for serotype 1, 2 and 3 respectively, were obtained with formulations containing sucrose and/or trehalose in combination with mannitol (Fig. 1b). Among the three serotypes, type 2 was the least affected during lyophilization, resulting in a maximum recovery of 85% after lyophilization. This serotype is known to be the most stable at higher temperatures (33).

This first experiment illustrates the complexity of lyophilizing a trivalent polio vaccine retaining its antigenicity and displays that each serotype may behave different within the same formulations. It has been reported earlier that lyophilization of polio vaccines is challenging. For example, Nagel *et al.* revealed that with the excipients sorbitol and peptone relative potencies of 70% for type 1 and 50% for type 2 could be obtained after lyophilization of IPV, while all activity of type 3 was destroyed (22). In addition, Pollard *et al.* exhibited that lyophilization of the wild type poliovirus resulted in almost complete inactivation (34), although it was not determined whether D-antigenicity was also negatively affected.

### Drying Methods

**Fig. 2:**
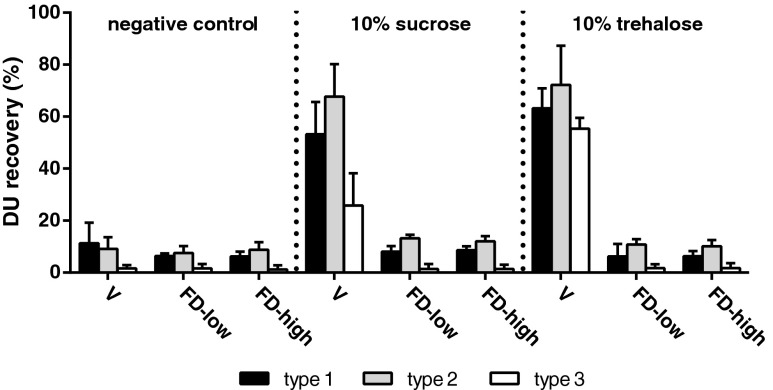
DU recovery of dried IPV using different drying methods, i.e., vacuum drying (V) or lyophilization with low (FD-low) or high freezing rate (FD-high). Common used stabilizing sugars sucrose (10% w/v) and trehalose (10% w/v) were compared with the formulation without additives (negative control). Mean values (*n* = 3) and SD are shown.

Literature indicates that vacuum drying, a drying process without a freezing step, can be used to stabilize IPV (35). With the use of disaccharides sucrose and trehalose as stabilizing agents, it is feasible to obtain a dried (or highly viscous) polio vaccine by vacuum drying without affecting its potency. As a result, we decided to investigate the impact of different drying processes on the integrity of IPV. Trehalose and sucrose based IPV formulations were vacuum dried and compared with the same formulations that underwent different lyophilization processes. One lyophilization process starting with a slow freezing step (cooling of shelves from 4°C to −50°C, at 1°C/min) and one with a fast freezing step (shelves pre-cooled to −50°C as has been used in the pilot experiment). The conventional trivalent IPV formulation, without any additives, showed recoveries <15% for all serotypes after vacuum drying or lyophilization (Fig. 2). The vacuum drying process yielded highly viscous IPV formulations with a water content of respectively 9% or 12% for the formulations containing sucrose or trehalose, whereas lyophilization yielded formulations with a water content of <1%. While more than 55% of the antigen is intact after vacuum drying of IPV containing 10% trehalose, both lyophilization processes resulted in almost complete loss of D-antigenicity. These results depict both one of the main disadvantages of vacuum drying as well as the opportunity to dry without freezing stresses. The high moisture content is caused by the relatively low specific surface area during vacuum drying, which results in an extremely slow secondary drying when compared to lyophilization. As a result, the risk of sugar crystallization and/or phase separation in the rubbery state exists (13).

The poor recoveries depicted in Fig. 2 suggest that neither trehalose nor sucrose alone was able to protect IPV against both the freezing and drying stresses during lyophilization. Although the different freezing rates of the lyophilization processes did not show significant differences in D-antigenicity, particle size measurements after freeze thawing revealed differences in IPV particle size that were dependent on freezing rate. Slowly frozen IPV had a size of 44.7 ± 2.1 nm with a PdI of 0.485 ± 0.062, whereas fast frozen IPV remains at a particle size of 38.7 ± 1.1 nm with a PdI of 0.166 ± 0.009 (similar to IPV bulk prior to freeze thawing). Thus, IPV appeared to be most resistant to freeze thawing when a fast freezing rate was applied, which induced less aggregation than slow freezing. For that reason, a fast freezing step was selected for optimization of the IPV formulation for lyophilization.

### Extensive Excipient Screening

In order to obtain an IPV formulation that is suitable for lyophilization we performed a more extensive excipient screening. The selection of excipients for the screening (Table I) was based on findings from literature (20,22,36–40) and earlier unpublished data.

**Table I:** Formulations Tested in an Excipient Screening Experiment (S). DU Recoveries were Determined Directly After Lyophilization

	Sugars	Polyols	Amino acids	Proteins	Other	DU recovery (%)		
						T1	T2	T3
S0	–	–	–	–	–	12	17	2
S1^a^	–	7% sorbitol 7% mannitol	2% MSG 2% glycine	7% ovalbumin	–	66	89	73
S2^a^		7% sorbitol 7% mannitol	2% MSG 2% glycine	7% ovalbumin	10 mM EDTA	66	81	77
S3^a^	–	7% sorbitol 7% mannitol	2% MSG 2% glycine	–	–	73	95	74
S4	3% sucrose 3% dextran 3% myo-inositol	–	–	3% ovalbumin	–	27	58	12
S5	5% sucrose	5% sorbitol 5% mannitol	2% glycine 3% lysine 3% arginine			15	49	5
S6^a^	5% sucrose	5% sorbitol 5% mannitol	3% MSG 2% glycine 3% lysine	–	–	65	86	76
S7	5% sucrose 5% trehalose		3% lysine 3% alanine			34	67	36
S8	5% sucrose 5% trehalose		3% lysine 3% alanine		0.01% Tween80	38	82	43
S9	5% sucrose		3% lysine 3% alanine		3% Ca-lactobionate	36	36	24
S10	5% sucrose		3% lysine 3% alanine		3% rec. gelatin	45	72	46
S11^a^	–	5% sorbitol	–	5% peptone	2% MgCl_2_	80	79	75
S12^a^	–	5% sorbitol	–	5% peptone	1% LiCl	86	100	87
S13	5% sucrose 5% trehalose	–	–	5% peptone	–	29	62	26

^a^Formulation selected for stability testing (as shown in Fig. 3)

**Fig. 3:**
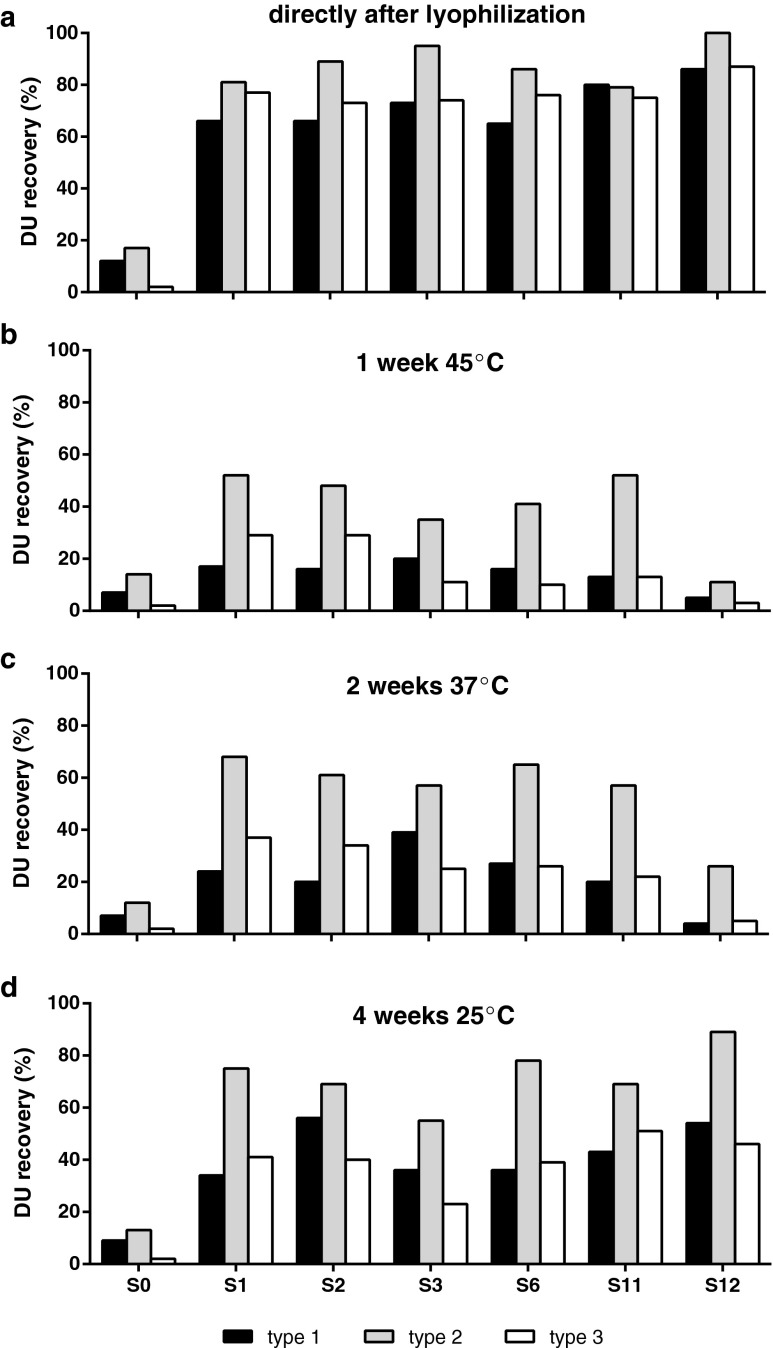
DU recoveries of the best formulations from the screening experiment based on recoveries directly after lyophilization (>60% for all serotypes). Panel (**a**) shows the DU recoveries directly after lyophilization. Panel (**b**, **c** and **d**) show the recoveries after incubation for 1 week at 45°C, 2 weeks at 37°C, and 4 weeks at room temperature, respectively. The formulations are described in Table I.

In general, the formulations containing sorbitol showed high recoveries directly after lyophilization. Especially formulations containing sorbitol, mannitol and monosodium glutamate (MSG) stabilized the IPV during the process of freezing and subsequent dehydration with DU recoveries of >65% for all serotypes (Table I; S1–S3, S6). Another notable formulation is the combination of sorbitol, peptone and the salts LiCl or MgCl_2_ (Table I; S11 and S12) indicating that this combination of excipients is able to protect the IPV during lyophilization. The best formulations, which showed DU recoveries of >60% directly after lyophilization for all serotypes, were selected and subsequently tested for stability (Fig. 3). In general, the tested formulations so far showed disastrous recoveries after incubation at higher temperatures (Fig. 3b–c) and even after incubation for a month at ambient temperature, a large drop in antigenicity was observed (Fig. 3d).

The appropriate performance of sorbitol combined with mannitol and MSG indicates that the presence of polyols in combination with MSG stabilizes the IPV during lyophilization in a similar way as the disaccharides sucrose and/or trehalose (combined with mannitol) did as shown in the pilot study. In earlier lyophilization studies, sorbitol was used as excipient in combination with peptone (22), which showed again to be a valuable combination in this experiment, whether or not in the presence of a salt like MgCl_2_ or LiCl. Peptone-containing lyophilized IPV formulations, however, showed limited storage stability at elevated temperatures (Fig. 3b–c). Furthermore, peptone is poorly defined and heterogeneous in composition; therefore, it was chosen to exclude peptone further in the formulation design. MgCl_2_’s stabilizing potential has been described for fluid oral polio vaccine. As a result, a number of manufacturers use MgCl_2_ to stabilize their OPV (39). Thus, MgCl_2_ could also be a critical additive in a dried IPV formulation.

### Design of Experiments—Full Factorial Design

Based on the results of our study so far, we selected the most promising excipients to screen them further using a DoE approach and get more insight in the IPV stabilizing potential of these additives. Therefore, a full factorial design was performed around the excipients sorbitol, MSG, MgCl_2_ and mannitol (all in the concentration range of 0–10% w/v) (Table II). The mixture of sorbitol, mannitol and MSG has already shown its capacity to provide valuable protection during the lyophilization process. In addition, mannitol was selected as a common bulking agent due to its excellent cake-forming property and the option to apply annealing when needed (41,42). As mentioned above, MgCl_2_ is already a proven stabilizer for OPV and therefore we examined its contribution in a dried IPV formulation as well.

**Table II:** Excipients Sorbitol, MgCl_2_, MSG and Mannitol (all 0–10% w/v) Examined in a Full Factorial Design (D). Glass Transition Temperature (T_g_’) of the Liquid Formulation Before Lyophilization and Residual Moisture Content (RMC) of the Dried Cake were Determined

	Sorbitol	MgCl_2_	MSG	Mannitol	T_g_’	RMC
					(°C)	(%)
D1	–	–	–	–	n.d.	0.2
D2	10%	–	–	–	−43.4	2.4
D3	–	10%	–	–	n.d.	45.9
D4	10%	10%	–	–	n.d.	20.6
D5	–	–	10%	–	−47.0	7.4
D6	10%	–	10%	–	−41.2	1.8
D7	–	10%	10%	–	−58.6	4.0
D8	10%	10%	10%	–	−49.6	7.6
D9	–	–	–	10%	−34.6	1.1
D10	10%	–	–	10%	−40.5	0.3
D11	–	10%	–	10%	n.d.	16.1
D12	10%	10%	–	10%	−51.6	17.1
D13	–	–	10%	10%	−41.0	2.8
D14	10%	–	10%	10%	−39.7	2.3
D15	–	10%	10%	10%	−51.2	8.2
D16	10%	10%	10%	10%	−48.2	9.7
D17	5%	5%	5%	5%	−48.1	5.8
D18	5%	5%	5%	5%	−47.7	17.7
D19	5%	5%	5%	5%	−47.7	9.7

**Fig. 4:**
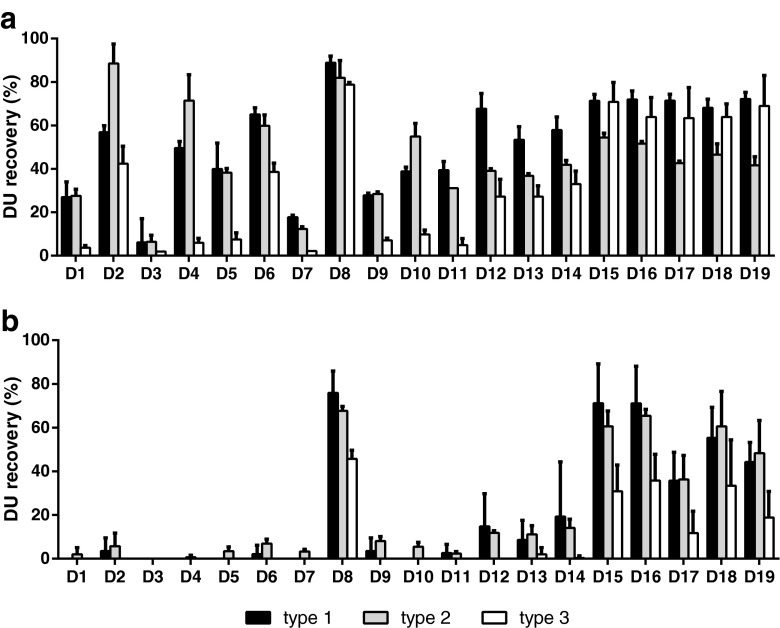
The stabilizing effect of sorbitol, MgCl_2_, MSG and mannitol was investigated in a screening experiment using a DOE approach. Mean DU recoveries and standard deviations (*n* = 3) directly after lyophilization (**a**) and after incubation for 1 week at 45°C (**b**) are shown.

**Fig. 5:**
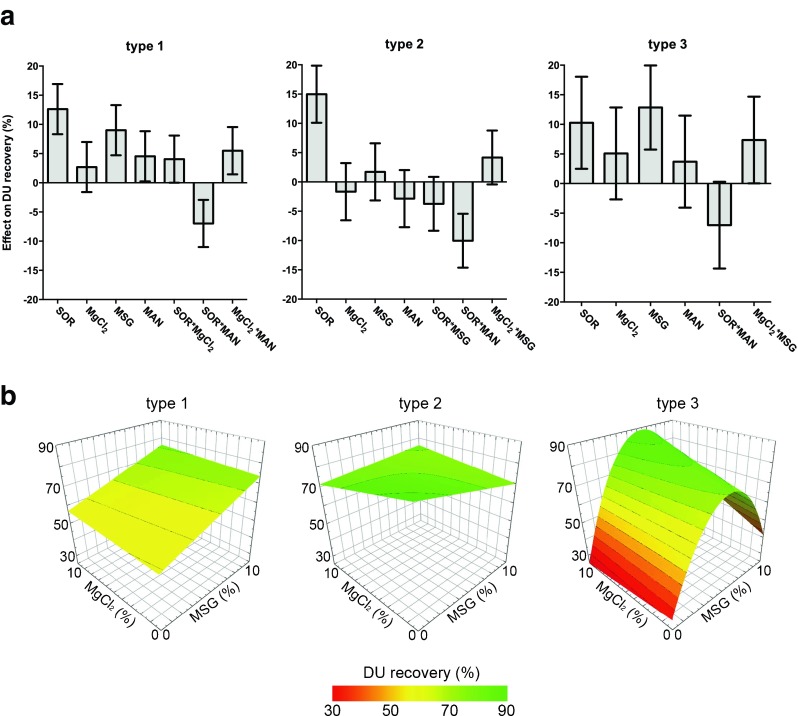
Stabilizing potential of the excipients sorbitol (SOR), MgCl_2_, monosodium glutamate (MSG) and mannitol (MAN) on the DU recovery directly after lyophilization. Main and interaction effects that contribute (per serotype) to the best fitted model, according to their model performance parameters (Q^2^ = 0.685, R^2^ = 0.923 (type 1); Q^2^ = 0.577, R^2^ = 0.877 (type 2); Q^2^ = 0.575, R^2^ = 0.824 (type 3)) are shown in coefficient plots (**a**). Surface response plots of the DU recovery for each serotype based on formulations containing MSG and MgCl_2_ in combination with 10% sorbitol (without mannitol) (**b**).

Figure 4a shows that sorbitol seemed to be an important excipient for the stabilization of both type 1 and 2 during lyophilization with recoveries up to 89% (Formulation D2 and D8). DU recoveries after one week accelerated stability testing clearly illustrate the benefit of sorbitol, as well as MgCl_2_ and MSG in the formulation (Fig. 4a, formulation D8). Regression models (R^2^ > 0.65 and Q^2^ > 0.5) of these data confirm the findings that are described above. The stabilizing effects of the excipients on DU recovery directly after lyophilization are depicted per serotype in coefficient plots after model optimization (Fig. 5a). For all serotypes, there is a main effect of sorbitol, whereas MSG showed to be significant beneficial for the DU recovery of type 1 and 3. However, sorbitol showed an interaction with mannitol that negatively affected IPV recovery after lyophilization, implying that the addition of mannitol to the IPV formulation for lyophilization is not desired. Accelerated stability testing revealed that MgCl_2_ and MSG are important stabilizers for all serotypes, indicated by significant main effects and interaction between these excipients. This means that the addition of MgCl_2_ or MSG (10% w/v) results in an increase of 7–13% in DU recovery, while the combination of these excipients would boost the D-antigenicity with 6–10%. The inclusion of MgCl_2_ to the IPV formulation increases the residual water content (Table II) after lyophilization significantly. This excipient is also responsible for reducing the glass transition temperatures (T_g_’) of the formulation before drying. The sorbitol and MSG interaction factor is able to increase the T_g_’ significantly (data not shown). As such, formulations might be optimized for T_g_’ by increasing the sorbitol-MSG content in order to be capable to lyophilize at higher temperatures resulting in shorter process time.

Again, it was observed that there are some divergences between the serotypes regarding their preference for stabilizers during lyophilization, whereas all serotypes seemed to prefer the presence of MSG and MgCl_2_ during stability testing. The surface response plots demonstrate that for type 1, the highest DU recoveries after lyophilization were found in a formulation containing 10% sorbitol in the presence of the highest amounts of MSG, regardless the MgCl_2_ concentration, whereas both additives have no significant effect on type 2 (Fig. 5b). Though, type 3 showed to be most delicate for small differences in excipient concentrations with maximal DU recoveries of more than 80% with a formulation containing 10% sorbitol, 5–10% MgCl_2_ and 4–8% MSG.

### Optimization

With the purpose to optimize the formulation, a response surface methodology design was implemented. Hence, a central composite circumscribed (CCC) (43) study set-up with the factors sorbitol (8–12% w/v), MSG (8–12% w/v) and MgCl_2_ (5–10% w/v) was designed (Table III) and DU recovery was determined directly after lyophilization. An extensive stability study was included here as well to test whether the dried IPV formulation has an improved stability when compared with the conventional liquid IPV.

**Table III:** Sorbitol (8–12% w/v), MgCl_2_ (5–12% w/v) and MSG (5–12% w/v) Tested in a Central Composite Circumscribed Design. DU Recoveries were Determined per Serotype Directly After Lyophilization and After Accelerated Stability Testing (one week at 45°C)

	Sorbitol	MgCl_2_	MSG	DU recovery (%)			DU recovery (%)		
				Directly after lyophilization			Stability 45°C		
				T1	T2	T3	T1	T2	T3
O1	8%	5%	5%	98.6	96.5	85.7	91.9	97.0	55.2
O2	12%	5%	5%	88.9	88.7	65.2	87.6	91.6	73.7
O3	8%	12%	5%	76.0	82.4	33.6	62.5	49.2	12.2
O4	12%	12%	5%	89.4	91.7	59.1	95.8	90.8	48.2
O5	8%	5%	12%	90.6	81.9	70.7	86.0	81.4	57.5
O6	12%	5%	12%	79.3	82.5	62.1	80.2	77.0	39.4
O7	8%	12%	12%	84.9	86.7	64.6	92.0	84.6	63.5
O8	12%	12%	12%	87.9	82.5	73.0	90.3	86.7	69.8
O9	6.6%	8.5%	8.5%	90.0	99.3	84.8	93.3	90.6	73.0
O10	13.4%	8.5%	8.5%	86.2	85.3	67.4	86.1	85.3	69.3
O11	10%	2.6%	8.5%	77.5	72.4	47.4	74.4	60.7	29.9
O12	10%	14.4%	8.5%	89.1	84.6	63.6	91.7	72.7	40.0
O13	10%	8.5%	2.6%	98.1	85.2	49.2	93.0	83.3	42.2
O14	10%	8.5%	14.4%	84.7	70.3	66.3	74.8	81.2	35.5
O15	10%	8.5%	8.5%	86.6	88.4	68.2	90.1	91.1	67.3
O16	10%	8.5%	8.5%	92.2	90.8	75.1	93.5	89.1	69.2
O17	10%	8.5%	8.5%	96.0	98.3	76.7	91.7	94.0	57.0

**Fig. 6:**
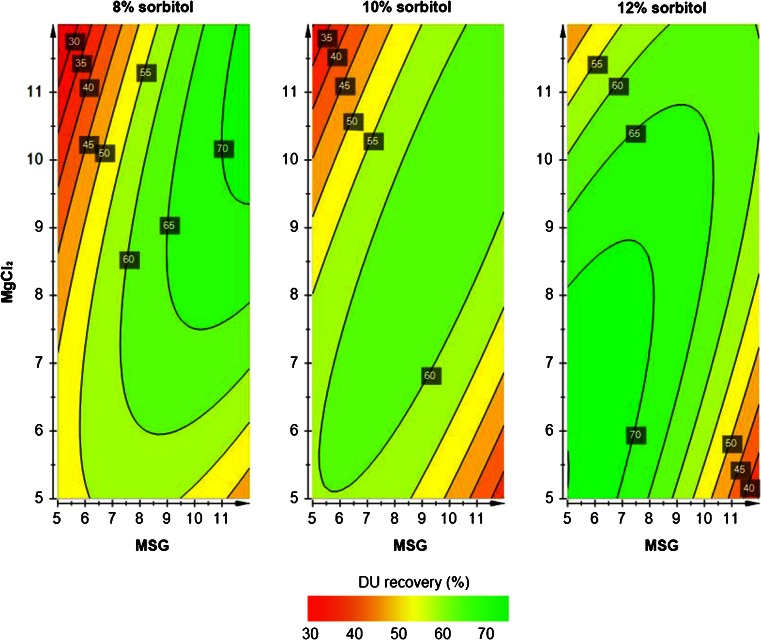
Contour plots show the effect of MSG (5–12% w/v) and MgCl_2_ (5–12% w/v) in combination with 8% (**a**), 10% (**b**) or 12% w/v sorbitol (**c**) on the DU recovery after lyophilization and subsequent accelerated stability testing for serotype 3.

Within the design of the study, all formulations resulted in comparable recoveries after lyophilization and subsequent storage (Table III). This indicates the formulation robustness of the formulation based on sorbitol (8–12% w/v), MSG (8–12% w/v) and MgCl_2_ (5–10% w/v). Due to these small differences in DU recovery after lyophilization and stability assessment between the tested formulations, it was not possible to obtain a valid model to describe the data. With all formulations, satisfying stabilization was achieved for serotypes 1 and 2, which showed recoveries of between 75% and 100% directly after lyophilization and only a small loss (0% to maximal 15%) during accelerated stability testing (Table III). As mentioned above, type 3 showed to be the determining aspect in the decision for the final concentrations of the three excipients in our IPV formulation. Therefore, we decided to focus on the stability data of this serotype. PLS regression describes the accelerated stability data (1-week incubation at 45°C) well with high values for model validity and reproducibility (respectively 0.67 and 0.86). However, the predicting power of the model is limited (Q^2^ = 0.43 instead of Q^2^ > 0.5). Despite this limitation, we requested the contour plots (Fig. 6) to get an indication of the important parameters to stabilize serotype 3. It seems that the best DU recoveries (>70%) were obtained with the lowest sorbitol (8% w/v) concentration in combination with the highest concentrations of both MgCl_2_ and MSG (>10% w/v) or the highest sorbitol (12% w/v) content combined with relatively low amounts of MgCl_2_ and MSG (<8% w/v) (Fig. 6).

**Fig. 7:**
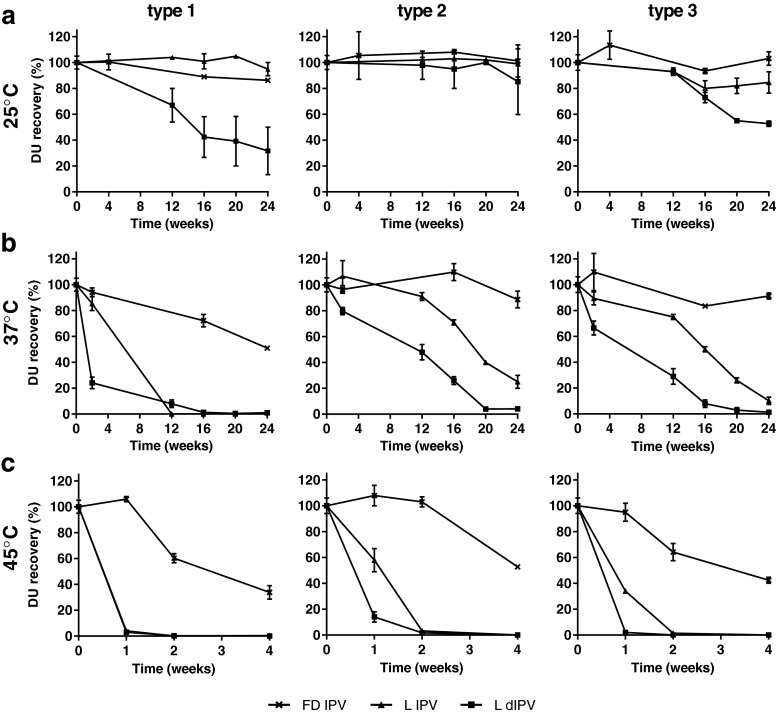
Stability testing of a lyophilized trivalent IPV formulation containing 10% (w/v) sorbitol, 8.5% (w/v) MgCl_2_ and 8.5% (w/v) MSG at 25°C (**a**), 37°C (**b**) or up to 4 weeks at 45°C (**c**). The freezedried formulation (FD IPV) was compared with the conventional liquid IPV (L IPV) and a liquid IPV dialyzed against McIlvaine buffer (L dIPV), which is the buffer of choice during lyophilization.

This experiment showed the robustness of the formulation since many formulations containing sorbitol, MgCl_2_ and MSG in the tested concentrations showed high recoveries directly after lyophilization and preserved antigenicity during accelerated stability. Based on excellent DU recoveries after lyophilization and subsequent stability testing (Table III), the formulation containing 10% (w/v) sorbitol, 8.5% (w/v) MgCl_2_ and 8.5% (w/v) MSG was selected for additional extensive stability testing up to 24 weeks at 25°C and 37°C, and up to 1 month at 45°C. Both the conventional liquid IPV and lyophilized IPV formulation remain stable during long-term incubation at ambient temperature (Fig. 7a). However, at elevated temperatures, the lyophilized IPV formulation revealed its improved stability profile. Despite its relatively high residual moisture content, which was determined at 4.5 ± 0.9%, minimal loss was observed for the dried IPV after storage at temperatures above room temperature where the liquid IPV has lost its antigenicity completely (Fig. 7b and c).

This study shows the feasibility to convert IPV into the dry state using lyophilization. The focus here was on development and optimization of a dried IPV formulation. However, the lyophilization process needs optimization as well, since the RMC of our lyophilized IPV formulation exceeds the limit of 3% water content from the European Pharmacopoeia. This high residual water content is probably due to the presence of MgCl_2_, a hexahydrate with strongly bound water, in the IPV formulation. The duration of the secondary drying step dictates the residual moisture level in a lyophilized product (20), so probably prolongation of this drying step and raising the end temperature of the lyophilization process could decrease the RMC and thus could possibly improve the final product.

## Conclusion

The aim of the study was to develop a dried IPV formulation with minimal loss during the drying process and improved stability when compared with the conventional liquid IPV, which could allow distribution and storage under unfrigerated conditions. Extensive screening of a large number of excipients combined with a DoE approach yielded a lyophilized IPV formulation with remaining antigenicity for all serotypes when kept at ambient or even higher temperatures.

Although further improvement and research is still possible, this study showed the potential of a highly stable and safe lyophilized polio vaccine, which could be distributed in developing countries without the need of a cold-chain transport.

## Electronic supplementary material

Below is the link to the electronic supplementary material.

Table SI (DOCX 20 kb)

## Abbreviations

(s)IPV: (Sabin) inactivated polio vaccine
cVDPV: Circulating vaccine-derived poliovirus
DoE: Design of experiments
DU: D-antigen unit
MSG: Monosodium glutamate
OPV: Oral polio vaccine
PLS: Partial least square
RMC: Residual moisture content
VAPP: Vaccine-associated paralytic poliomyelitis
