# Supplementary material for: Development of Thermostable Lyophilized Inactivated Polio Vaccine
Source: Pharm Res. 2014 Apr 24;31(10):2618–29. doi: 10.1007/s11095-014-1359-6 (PMC4197379; doi:10.1007/s11095-014-1359-6)
Supplement: Supplementary file 1 — (DOCX 20 kb) [file 11095_2014_1359_MOESM1_ESM.docx]

Table S1

Commonly used excipients tested using a design of experiments approach. DU recoveries were determined per serotype by ELISA directly after lyophilization.

|  | Sucrose | Trehalose | Mannitol | Dextran | NaCl (mM) | DU recovery (%) | | |
| --- | --- | --- | --- | --- | --- | --- | --- | --- |
|  |  |  |  |  |  | **T1** | **T2** | **T3** |
| P1 | - | - | - | - | - | 8.5 | 11.3 | 1.8 |
| P2 | 20% | 20% | - | - | - | 30.3 | 58.0 | 22.5 |
| P3 | 20% | - | 10% | - | - | 45.3 | 68.3 | 46.4 |
| P4 | - | 20% | 10% | - | - | 44.4 | 68.5 | 40.5 |
| P5 ^a^ | 11.4% | 11.4% | 5.7% | 5.7% | - | 30.5 | 51.1 | 29.9 |
| P6 | - | - | 6.7% | 10% | - | 25.9 | 54.1 | 8.9 |
| P7 | - | 6.7% | 10% | 10% | - | 31.6 | 62.2 | 16.3 |
| P8 ^a^ | 13.3% | 13.3% | 2.2% | 6.7% | - | 33.9 | 57.8 | 25.9 |
| P9 | 6.7% | - | 10% | 10% | - | 34.9 | 58.1 | 27.5 |
| P10 | 10% | 10% | - | 5% | - | 22.2 | 45.0 | 8.2 |
| P11 | 10% | 10% | 5% | - | - | 35.5 | 62.7 | 34.9 |
| P12 | 20% | - | - | - | 63 | 32.8 | 59.3 | 33.3 |
| P13 | - | 20% | - | - | 63 | 21.7 | 47.6 | 7.1 |
| P14 | - | - | 10% | - | 63 | 39.8 | 57.9 | 44.2 |
| P15 | - | - | - | 10% | 63 | 8.2 | 15.1 | 4.0 |
| P16 | 10% | - | 5% | 5% | 31.5 | 31.9 | 56.7 | 31.5 |
| P17 | 10% | 10% | 5% | 5% | 63 | 29.2 | 49.6 | 25.2 |
| P18 | 16% | 16% | 8% | - | 50 | 42.3 | 64.6 | 42.0 |
| P19 ^a^ | 13.3% | 13.3% | - | 6.7% | 41.7 | 22.7 | 43.7 | 9.9 |
| P20 ^a^ | 11.4% | - | 5.7% | 5.7% | 35.7 | 33.1 | 56.3 | 32.9 |
| P21 | - | 16% | 8% | 8% | 50 | 25.9 | 49.1 | 16.2 |
| P22 ^a^ | 13.3% | 6.7% | 3.3% | 3.3% | 41.7 | 29.2 | 52.2 | 27.2 |
| P23 | 10% | 10% | 5% | 5% | 31.5 | 29.2 | 49.6 | 25.2 |
| P24 | 10% | 10% | 5% | 5% | 31.5 | 30.3 | 52.8 | 27.2 |
| P25 | 10% | 10% | 5% | 5% | 31.5 | 34.0 | 58.1 | 28.1 |

^a^ Concentrations differ from design due to maximal solubility of the formulation
